# Catheter-related deep vein thrombosis complicated by iatrogenic pulmonary embolism after thrombectomy leading to hemodialysis failure and successful rescue by automated peritoneal dialysis: A case report

**DOI:** 10.1097/MD.0000000000051337

**Published:** 2026-10-30

**Authors:** Wenhui Lu, Rui Gong, Xi Liu, Chengyin Yang, Ruixue Li, Hui Yang

**Affiliations:** a Department of Nephrology, Chongqing Yubei People’s Hospital, Chongqing, China; b Department of Oncology and Hematology, Chongqing Yubei People’s Hospital, Chongqing, China.

**Keywords:** automated peritoneal dialysis, catheter-related thrombosis, Elderly ESRD, hemodialysis, pulmonary embolism

## Abstract

**Rationale:**

Catheter-related deep vein thrombosis (DVT) is a serious complication in hemodialysis (HD) patients and may lead to pulmonary embolism (PE). Interventional treatment of catheter-related DVT may be complicated by acute PE and other life-threatening events. When recurrent vascular access failure and cardiovascular intolerance make HD unsustainable, automated peritoneal dialysis (APD) may provide an alternative renal replacement strategy. We report a case of successful rescue with APD after severe complications following interventional treatment of catheter-related DVT.

**Patient concerns:**

An 82-year-old woman with end-stage renal disease developed right femoral vein thrombosis shortly after initiating urgent-start HD via a femoral catheter. Following percutaneous mechanical thrombectomy and catheter-directed thrombolysis, she developed acute hypoxemia, pulmonary embolism, severe thrombocytopenia, heart failure, and gastrointestinal bleeding. Recurrent central venous catheter dysfunction and repeated hypotension and oxygen desaturation during HD made continued conventional HD increasingly difficult.

**Diagnoses:**

The patient was diagnosed with catheter-related DVT complicated by acute PE following interventional thrombectomy.

**Interventions:**

The patient received intensive supportive care, including anticoagulation when clinically feasible, continuous renal replacement therapy, blood transfusions, and management of gastrointestinal bleeding and heart failure. Because of recurrent central venous catheter dysfunction and intolerance to conventional HD, a multidisciplinary team recommended peritoneal dialysis (PD). After clinical stabilization and PD catheter placement, she was transitioned to APD.

**Outcomes:**

After APD initiation, the patient’s uremic symptoms and lower-extremity edema resolved, and her functional capacity improved from bedridden to independent walking. Hemoglobin increased from 65.7 to 118.2 g/L, cardiac function improved, and adequate dialysis was achieved with a total dialysis adequacy index of 2.58. During approximately 10 months of follow-up, she remained free from peritonitis and PD catheter dysfunction.

**Lessons:**

This case highlights the risk of serious thromboembolic complications following interventional treatment of catheter-related DVT in HD patients. When recurrent vascular access failure and cardiovascular intolerance render conventional HD unsustainable, timely reassessment of dialysis modality is crucial; APD may offer an effective rescue strategy for selected elderly patients.

## 1. Introduction

The selection of dialysis modality for end-stage renal disease (ESRD) patients involves balancing clinical risks and quality of life. Hemodialysis (HD) remains the most widely used modality,^[1]^ but it requires reliable vascular access. central venous catheter (CVC) are frequently used for urgent-start HD; however, catheter-related deep vein thrombosis (DVT) is a common and serious complication.^[2–4]^ DVT can lead to pulmonary embolism (PE), and the risk of PE is higher in HD patients than in peritoneal dialysis (PD) patients (adjusted HR 2.30, 95% CI 1.23–4.29).^[5]^ When DVT occurs with limb-threatening symptoms, interventional procedures such as percutaneous mechanical thrombectomy (PMT) or catheter-directed thrombolysis (CDT) may be necessary.^[6]^ Nonetheless, these procedures themselves carry a risk of embolization and iatrogenic PE.^[7]^

Peritoneal dialysis, particularly automated peritoneal dialysis (APD), offers advantages in preserving residual renal function, maintaining hemodynamic stability, and avoiding vascular access-related complications.^[8]^ APD has also been associated with lower all-cause mortality risk compared to continuous ambulatory PD (HR 0.87, 95% CI 0.77–0.99),^[9]^ as well as lower peritonitis rates and better psychosocial acceptance.^[10]^ For patients who develop catastrophic complications from HD vascular access, a switch to PD can be a lifesaving strategy.^[11]^

Here, we present an 82-year-old female ESRD patient who developed right femoral vein thrombosis shortly after HD initiation via a femoral CVC. Following interventional thrombectomy, she suffered acute PE complicated by respiratory failure, severe thrombocytopenia, and recurrent CVC dysfunction, ultimately requiring transition to APD. This case highlights the risks of HD catheter-related interventions and the value of APD as a rescue therapy.

## 2. Case presentation

An 82-year-old woman with a 20-year history of hypertension and coronary artery disease, a remote history of atrial septal defect repair (5 years previously), and a remote history of cerebral infarction over 20 years ago without residual deficits, presented to our hospital on January 31, 2025, with a 2-day history of right lower limb swelling. She had been diagnosed with chronic kidney disease stage 5 2 years earlier and had recently initiated urgent-start hemodialysis (HD) via a right femoral vein CVC on January 8, 2025, due to progressive dyspnea. After initial improvement, she was discharged on January 28, 2025. Two days later, she developed painless swelling of the right lower limb, which rapidly progressed from the calf to the thigh.

On admission, her vital signs were stable: temperature 36.9°C, pulse 75 beats/minutes, respiratory rate 20 breaths/minutes, and blood pressure 141/73 mm Hg. Physical examination revealed a chronic kidney disease facies and moderate pallor. The right lower limb was markedly swollen compared to the left, with pitting edema predominating in the calf. The right popliteal and femoral arteries were palpable, but the right dorsalis pedis artery was faintly palpable. Laboratory findings were notable for: hemoglobin 100 g/L, platelet count 50 × 10^9^/L, serum creatinine 330.92 μmol/L, D-dimer 24.82 μg/mL (markedly elevated). Coagulation profile showed a prolonged activated partial thromboplastin time (>400.0 seconds). Right lower extremity venous ultrasound confirmed DVT at the site of the femoral catheter. Chest computed tomography (CT) revealed bilateral pulmonary infiltrates (right middle lobe predominance), moderate right pleural effusion, and multiple pulmonary nodules. Because pulmonary nodules and infiltrative changes can also be observed in septic PE, infectious causes were considered during the subsequent evaluation (Figure 1A)Transthoracic echocardiography showed a left ventricular ejection fraction of 59%, left atrial enlargement, mild valvular regurgitation, and an estimated pulmonary artery systolic pressure of approximately 37 mm Hg. No valvular vegetation or McConnell’s sign was identified. The RV/LV ratio was not available from the original echocardiographic report.

**Figure 1.**
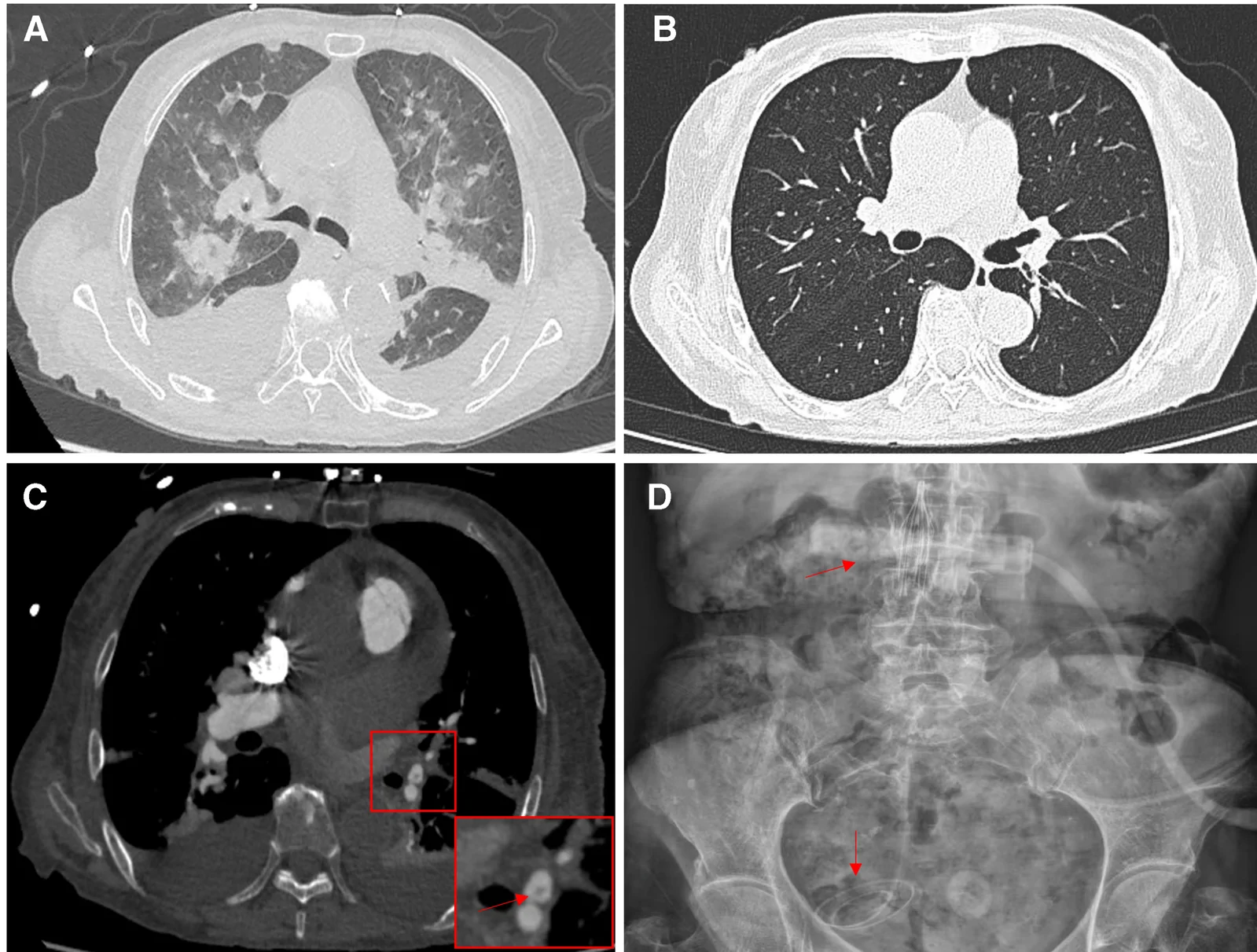
Representative imaging findings. (A) Chest computed tomography (CT) performed on February 4, 2025, demonstrating bilateral pulmonary infiltrates and right pleural effusion. (B) Follow-up chest CT performed on November 25, 2025, demonstrating near-complete resolution of the pulmonary abnormalities. (C) Contrast-enhanced chest CT performed on February 4, 2025, demonstrating a small pulmonary arterial filling defect (arrow), consistent with acute pulmonary embolism. (D) Abdominal radiograph obtained on May 16, 2025, showing an inferior vena cava filter (upper arrow) and a well-positioned peritoneal dialysis catheter (lower arrow).

On the day of admission (January 31, 2025), the patient underwent inferior vena cava (IVC) filter placement, right lower extremity venography, and PMT with CDT under local anesthesia. The right femoral CVC was removed, and a temporary left femoral CVC was placed. Immediately after the procedure, the patient developed acute hypoxemia and dyspnea. Her platelet count dropped sharply from 50 × 10^9^/L to 18 × 10^9^/L, and D-dimer decreased from 24.82 μg/mL to 4.52 μg/mL. Given the recent DVT and clinical deterioration, PE was suspected. Contrast-enhanced chest CT performed on February 4, 2025, demonstrated a small pulmonary arterial filling defect consistent with acute PE (Fig. 1C).Given the presence of pulmonary infiltrates and elevated inflammatory markers, septic PE was considered in the differential diagnosis. The patient was afebrile, and blood cultures and sputum cultures were negative. Tuberculosis-related examinations showed no abnormalities. Transthoracic echocardiography revealed no valvular vegetation or evidence of infective endocarditis. Although inflammatory markers were elevated, with a C-reactive protein level of 112.59 mg/L and a procalcitonin level of 0.948 ng/mL, the available microbiological and echocardiographic findings provided insufficient evidence for an infectious source of PE. Catheter-tip culture was not performed because there was no fever or clinical evidence strongly suggestive of catheter-related bloodstream infection. Therefore, septic PE was considered less likely but could not be completely excluded.

The patient was immediately transferred to the intensive care unit with a diagnosis of type I respiratory failure, acute heart failure, pulmonary infection, and bilateral pleural effusion. The left femoral catheter was nonfunctional and was removed; a right internal jugular vein CVC was inserted to initiate continuous renal replacement therapy. Mechanical ventilation, empirical meropenem for infection, and recombinant human interleukin-11 for thrombocytopenia were administered. Because of severe thrombocytopenia, peripheral blood smear examination was performed to evaluate possible thrombotic microangiopathy and pseudothrombocytopenia. No significant schistocytes or platelet clumping were observed, and these findings did not support thrombotic microangiopathy or pseudothrombocytopenia. Bone marrow examination was recommended to further evaluate the cause of thrombocytopenia; however, the patient and her family declined the procedure. Thromboelastography was also monitored and showed no significant abnormality. After clinical improvement, she was transferred back to the nephrology ward for conventional HD.

However, 5 minutes after starting conventional HD, she experienced sudden desaturation, hypotension, and bradycardia. A type A dialyzer reaction was suspected. Despite prompt treatment with atropine, epinephrine, and dexamethasone, she required endotracheal intubation and was re-admitted to the intensive care unit. After a course of invasive mechanical ventilation and continued antibiotic therapy, she improved and was again transferred back to the nephrology ward.

In the nephrology ward, she received intermittent bedside continuous renal replacement therapy, infection control, and supportive care. On March 16, 2025, she developed gastrointestinal bleeding, requiring cessation of rivaroxaban, fasting, intravenous proton pump inhibitors (esomeprazole), somatostatin, nutritional support, and multiple transfusions of red blood cells and plasma. After the bleeding resolved, her right internal jugular CVC once again became nonfunctional and required exchange over a guidewire. Given the recurrent thrombosis and catheter dysfunction, autoimmune and antiphospholipid-associated prothrombotic disorders were considered. Antiphospholipid antibodies were negative. Previous autoimmune testing performed in November 2024, including antinuclear antibody (ANA), anti-double-stranded DNA antibody (anti-dsDNA), complement C3, and complement C4, showed no abnormalities. The patient had not received any vaccination during the 1 to 2 months preceding the thrombotic event. However, a comprehensive thrombophilia evaluation was not available. This recurrent catheter dysfunction, combined with her intolerance to conventional HD, made continued HD increasingly challenging.

Given the patient’s intolerance to HD and the lack of a durable vascular access, a multidisciplinary team recommended PD. After optimizing nutrition, correcting anemia, and stabilizing metabolic status, she underwent laparoscopic PD catheter placement on April 24, 2025. She was initially managed with continuous ambulatory peritoneal dialysis (CAPD) and then transitioned to APD. She was successfully discharged home on APD.

### 2.1. Follow-up and outcome

One month after discharge on APD, the patient‘s uremic symptoms (nausea, fatigue) had resolved, and lower limb edema subsided. Her functional capacity improved from being bedridden to walking independently. Abdominal X-ray film dated May 16, 2025: The PD catheter is well positioned (Fig. 1D). A peritoneal equilibration test on May 22, 2025, showed high-average transport status, with a total dialysis adequacy index (Kt/V) of 2.58 (peritoneal Kt/V 2.13, residual renal Kt/V 0.46). Nutritional markers improved: hemoglobin recovered from a nadir of 65.7 g/L post-intervention to 118.2 g/L at last follow-up (Fig. 2A), and platelet count normalized (Fig. 2B). D-dimer decreased from 24.82 μg/mL to a minimum of 0.8 μg/mL (Fig. 2C). Serum albumin remained stable (Fig. 3B), and serum creatinine levels progressively decreased (Fig. 3A). Cardiac function improved to New York Heart Association class II-III, with left ventricular ejection fraction rising from 50% to 74% (Fig. 3C). Repeated chest CT on November 25, 2025, showed near-complete resolution of pulmonary infiltrates and pleural effusion (Fig. 1B).She has remained free from peritonitis or catheter dysfunction through the last follow-up.

**Figure 2.**
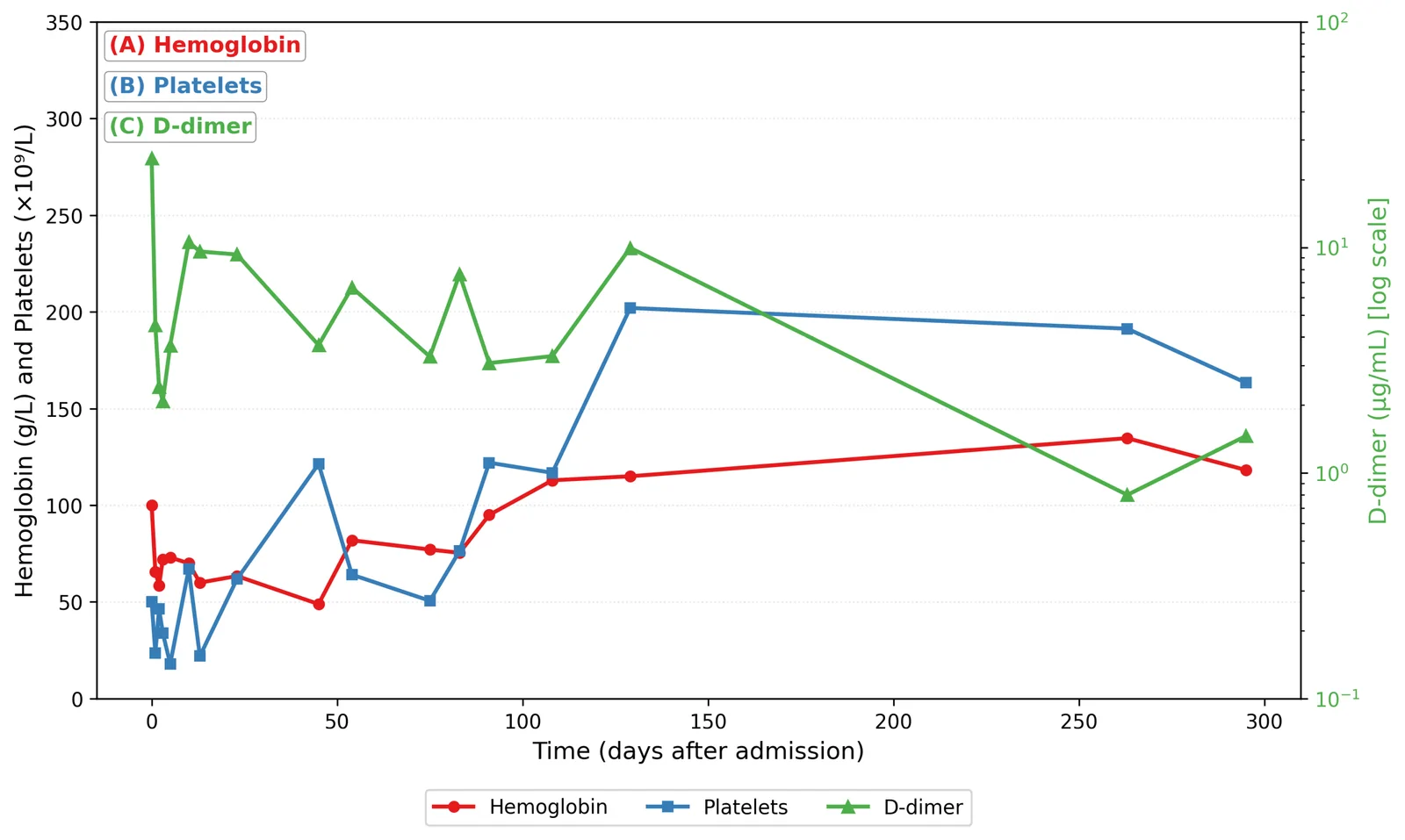
Longitudinal trends of blood parameters during hospitalization and outpatient follow-up. (A) Hemoglobin (g/L). (B) Platelet counts (×10^9^/L). (C) D-dimer levels (μg/mL, logarithmic scale). All panels share the same x-axis (days after admission).

**Figure 3.**
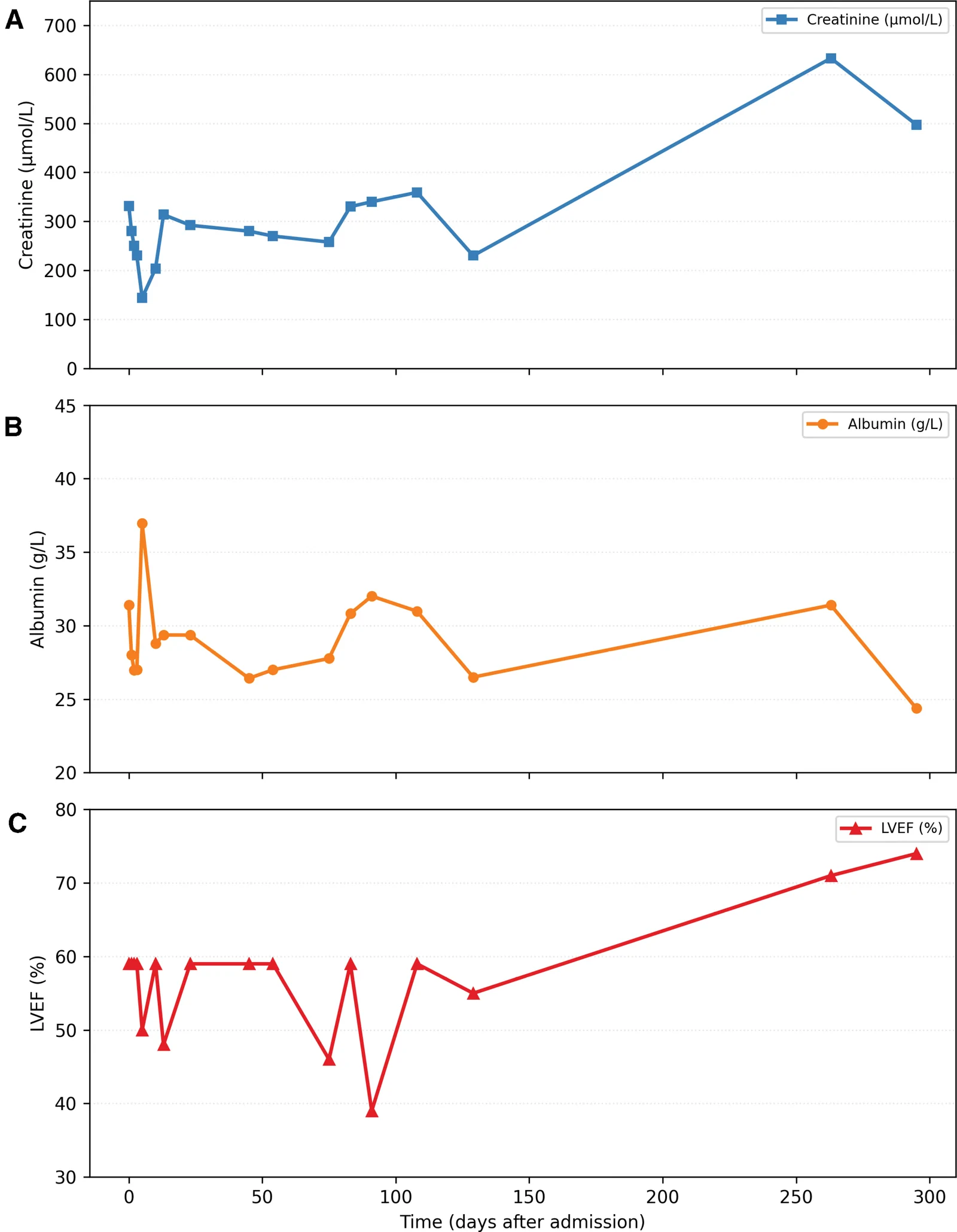
Longitudinal trends of metabolic and cardiac parameters during hospitalization and outpatient follow-up. (A) Serum creatinine (μmol/L). (B) Serum albumin (g/L). (C) Left ventricular ejection fraction (%). All panels share the same x-axis, days after admission.

## 3. Discussion

We report a case of an elderly HD patient who developed catheter-related DVT, suffered iatrogenic PE after interventional thrombectomy, and subsequently experienced recurrent CVC failure and HD intolerance, ultimately achieving a successful transition to APD. Several aspects of this case merit further discussion.

### 3.1. Interventional thrombectomy and iatrogenic PE

Lower extremity DVT with severe symptoms often warrants PMT or CDT to restore venous patency.^[6]^ Endovascular treatment facilitates thrombus removal in selected patients with lower extremity DVT, although PE remains a clinically important complication of DVT.^[7]^ In our patient, the acute onset of hypoxemia and thrombocytopenia immediately after the procedure, together with CT evidence of a small pulmonary arterial filling defect consistent with acute PE, raised the possibility of procedure-related embolization of thrombotic material during mechanical thrombectomy or thrombolysis. Although an IVC filter was placed, it may not capture fragments smaller than the filter pore size. This complication, while rare, should be anticipated in high-risk patients.

### 3.2. Platelet decline: consumption vs HIT

The marked decline in platelet count from 50 to18 × 10^9^/L within 24 hours after the procedure warrants careful consideration. In patients undergoing HD, thrombocytopenia may arise from multiple etiologies.^[12]^ Pulmonary embolism (PE) itself may lead to transient platelet consumption through activation of the coagulation cascade. Another important differential diagnosis is heparin-induced thrombocytopenia (HIT). The 4T scoring system, which assesses the severity of thrombocytopenia, timing of platelet decline, presence of thrombosis, and alternative causes of thrombocytopenia, is a validated clinical tool for HIT risk stratification.^[13]^ In the present patient, the immediate onset of thrombocytopenia following a single low-dose intraprocedural exposure to unfractionated heparin, together with rapid platelet recovery after heparin discontinuation, argues against immune-mediated HIT, which typically develops 5 to 10 days after an initial heparin exposure.^[14]^ Retrospective application of the 4T score indicated a low probability of HIT (3 points: moderate thrombocytopenia, platelet decline occurring within < 24 hours, and thrombosis attributable to preexisting DVT/PE as an alternative explanation). Although HIT antibody testing was not performed, representing a limitation of this report, the overall clinical presentation is more consistent with consumptive coagulopathy secondary to PE, potentially exacerbated by thrombolysis-associated platelet dysfunction.

### 3.3. Comparison with previous cases of HD-to-PD transition

To our knowledge, there is limited clinical evidence regarding switching dialysis modality from HD to PD in patients with intractable catheter-associated infection or exhausted vascular access. Ghandour et al successfully converted an ESRD patient with metastatic catheter-related bloodstream infection from intermittent HD to PD, highlighting the clinical role of PD under such clinical scenarios.^[15]^ However, that case did not involve PE or interventional thrombosis management. El-Reshaid et al described an HD patient switched to CAPD due to severe hemolysis from a traumatic aortic valve prosthesis.^[16]^ Compared with these reports, our case adds the unique features of iatrogenic PE following interventional thrombectomy for catheter-related DVT, severe thrombocytopenia requiring transfusion, recurrent HD intolerance (hypotension and desaturation) after CVC exchange, and successful rescue with APD rather than CAPD. The advanced age (82 years) and the sequence of multiple life-threatening complications before stabilization on APD distinguish this case from previously published transitions.

### 3.4. Challenges of continuing HD

After surviving the acute PE, the patient faced 2 major obstacles to ongoing HD: recurrent CVC dysfunction (both femoral and jugular catheters failed repeatedly, likely due to thrombosis or poor flow) and cardiovascular intolerance (hypotension and desaturation occurring minutes into HD sessions). These phenomena reflect the “vicious cycle” of HD dependence in a fragile patient with limited vascular access options and underlying cardiac compromise. Recent cohort data indicate that venous thromboembolism occurs at an incidence rate of 1.7 per 100 person-years in maintenance HD patients, with a 30-day case fatality rate of 20.8%.^[17]^ Moreover, both the patient and her family developed a strong psychological aversion to further conventional HD sessions, refusing to attempt them again. In such a scenario, switching to PD is not just an alternative but a necessary rescue therapy.^[11]^

### 3.5. Why APD succeeded

Peritoneal dialysis avoids the need for CVCs, thereby eliminating the risk of catheter-related thrombosis and bloodstream infection. APD, in particular, offers additional benefits over CAPD. A meta-analysis including over 230,000 patients showed that APD was associated with a significantly lower all-cause mortality risk compared to CAPD (HR 0.87, 95% CI 0.77–0.99), with particularly pronounced benefits in Asian populations (HR 0.76, 95% CI 0.67–0.86)^[9]^. APD also offers lower peritonitis rates, fewer mechanical complications (e.g., leaks, hernias), better ultrafiltration control, and enhanced quality of life due to nocturnal automated exchanges ^[10]^. Our patient, an 82-year-old woman with limited mobility and social support, was able to perform APD at home with assistance. Her clinical improvement resolution of uremic symptoms, correction of anemia, stabilization of cardiac function, and return to independent walking underscores the transformative potential of APD when HD is no longer feasible.

### 3.6. Clinical implications and limitations

This case illustrates that vascular access complications associated with HD can rapidly escalate and that timely transition to APD should be considered even after major adverse events. However, several limitations should be acknowledged. First, this is a single case report, which limits the generalizability of the findings. Second, the mechanism underlying the thrombocytopenia could not be definitively confirmed because HIT antibody testing was unavailable, although clinical assessment using the 4T score indicated a low probability of HIT. Bone marrow examination was considered but declined by the patient and her family, which limited further evaluation of the cause of thrombocytopenia. Third, the recurrent catheter dysfunction and thrombotic events raise the possibility of an underlying prothrombotic tendency. Antiphospholipid antibodies were negative, and previous autoimmune testing performed in November 2024, including ANA, anti-dsDNA, C3, and C4, showed no abnormalities. In addition, peripheral blood smear examination revealed no schistocytes or platelet clumping, providing no evidence suggestive of thrombotic microangiopathy or pseudothrombocytopenia. However, a comprehensive thrombophilia evaluation, including inherited and other acquired prothrombotic conditions, was not available. Therefore, the specific mechanism underlying the recurrent catheter dysfunction and thrombotic events could not be determined, and an underlying prothrombotic tendency could not be completely excluded. Fourth, the follow-up duration (approximately 10 months) was relatively short. Long-term outcomes, including peritonitis, technique survival, and patient survival, require further observation.

## 4. Conclusion

In conclusion, interventional treatment of HD catheter-related DVT carries a risk of iatrogenic PE, even with IVC filter placement. When HD becomes unsafe or impossible due to recurrent vascular access failure and cardiovascular instability, timely transition to APD can be a life-saving and effective strategy. This case supports the consideration of APD as rescue therapy in carefully selected ESRD patients who encounter catastrophic HD complications.

## Acknowledgments

The authors express their gratitude to the patient and her family for their cooperation.

## Author contributions

**Data curation:** Wenhui Lu, Chengyin Yang.

**Methodology:** Rui Gong.

**Validation:** Rui Gong, Xi Liu.

**Formal analysis:** Xi Liu, Ruixue Li.

**Supervision:** Ruixue Li.

**Writing – review & editing:** Hui Yang.

**Writing – original draft:** Wenhui Lu.
